# Associations between Chinese college students’ anxiety and depression: A chain mediation analysis

**DOI:** 10.1371/journal.pone.0268773

**Published:** 2022-06-02

**Authors:** Li-ying Wen, Liu-xia Shi, Li-jun Zhu, Meng-jie Zhou, Long Hua, Yue-long Jin, Wei-wei Chang

**Affiliations:** 1 Department of Epidemiology and Health Statistics, School of Public Health, Wannan Medical College, Wuhu, China; 2 Department of Oral Medicine, School of Stomatology, Wannan Medical College, Wuhu, China; Yamaguchi University: Yamaguchi Daigaku, JAPAN

## Abstract

**Objective:**

Anxiety and depression are great public health concerns among college students. The purpose of this study was to explore whether sleep quality and quality of life (QoL) play mediating roles in anxiety and depression among Chinese college students.

**Method:**

A total of 2757 college students (mean age = 19.07; SD = 1.14) completed the questionnaires, including a brief demographic survey. The 2-item General Anxiety Disorder (GAD-2) and the 2-item Patient Health Questionnaire (PHQ-2) were used to assess the symptoms of anxiety and depression, respectively. And the Pittsburgh Sleep Quality Index (PSQI) and the Short-Form 36 Health Survey (SF-36) were used to evaluate college students’ sleep quality and QoL, respectively. Mediation analyses were conducted by using PROCESS macro in the SPSS software.

**Result:**

Anxiety had both direct and indirect effects on depression. Sleep quality and QoL were not only independent mediators in the relationship between anxiety and depression but also chain mediators.

**Conclusion:**

The results of the current study highlight the crucial role of early intervention for depression with a focus on college students with anxiety, more especially, on those with poorer sleep quality and lower QoL.

## Introduction

Depression and anxiety are important indicators of mental health and have become the main risk factors of physical and mental health in the 21st century. The Global Burden of Diseases, Injuries, and Risk Factors 2019 study showed that the two most disabling mental disorders are depression and anxiety. Among adolescents, depression and anxiety disorders were ranked fourth and sixth in the list of leading causes of burden worldwide in 2019 [1]. This proves that depression and anxiety are prevalent and significant psychiatric disorders among young adults [2–5].

Chinese college students have unique characteristics and face different pressures from other groups. Some of the challenges they face are related to acquiring knowledge and gaining skills in college; cultivating intimacy during college, avoiding loneliness, and experiencing the realization of love; and adapting to college life. Since the reasons behind why they may suffer from anxiety and depression are different from that of other populations, this research specifically focus on the mental health of this group.

Depression and anxiety have profound negative implications on college students. First, depression and anxiety adversely affect academic performance [6–9], thereby resulting in increased college dropout rates [10–12]. Second, college students who experience depression and anxiety may not be able to properly cope with interpersonal relationships [13–15], such as establishing friendly peer relationships, maintaining harmonious family relationships, and upholding good teacher-student relationships. Third, a cross-sectional study of medical students predicted that the prevalence of major depressive disorders is associated with the presence of chronic diseases and major life events [16]. Another systematic review showed that anxiety among nursing students can have adverse effects on motivation, communication, learning, clinical practice, and the overall academic performance, which can result in their causing harm to patients [17]. Moreover, depression among college students is associated with physical illness, decreased physical activity, and increased levels of smoking [18] and substance abuse [19]. Furthermore, depression and anxiety have been found to increase the risk of suicidal thoughts and behaviors [20, 21]. A previous study on Chinese college students found that moderate-to-severe depression was positively correlated with declining lung function [22]. Given the substantial burden of depression and anxiety on individuals and society, it is crucial to understand college students’ development to promote preventive strategies and treatment.

Previous studies on anxiety and depression among college students have primarily focused on the prevalence, influencing factors, and possible adverse effects of such disorders. However, relatively few studies have observed the relationship between anxiety and depression. A cross-sectional study conducted among 4882 Chinese medical college students demonstrated a significant association between the two [23]. Moreover, previous studies on the relationship between social anxiety disorder and major depressive disorder have consistently represented a temporal precedence for social anxiety disorder relative to major depressive disorder [24, 25]. Therefore, in its exploration of the association and underlying mechanisms of anxiety and depression among college students, the current study assumes that there exists a time sequence relationship between them.

Additionally, anxiety can be used as a predictor of sleep quality, since students with anxiety are more likely to experience sleep problems [26–28]. One study showed that anxiety may affect sleep quality through the gray matter volume of the right insula [28]. Additionally, sleep quality is known to be correlated with depression among young adults, particularly college students [26, 28]. Several studies have found poor sleep quality to be correlated with remarkably high levels of depression among college students [29–32]. Furthermore, a longitudinal observational study found a bidirectional relationship between the Pittsburgh Sleep Quality Index (PSQI) global score and mental health problems, and found that baseline anxiety scores were correlated with the PSQI global score. The same study also showed that poorer baseline sleep quality was positively associated with depression, which provides some evidence for the relationship between anxiety symptoms and sleep quality, as well as the relationship between sleep quality and the development of depression [30]. The hypothalamic-pituitary-adrenal (HPA) axis may be a reason why sleep quality can act as a mediator of college students’ symptoms of anxiety and depression, since it regulates the levels of cortisol, adrenocorticotropic hormone, and corticotropin releasing hormone [33]. Additionally, there are 5-HT neurons located in the dorsal raphe nucleus project to the hypothalamus, and 5-HT is an excitatory neurotransmitter of corticotropin releasing hormones, which can excite the pituitary adrenocortical system [34]. Researches have predicted an association between 5-HT, sleep, anxiety, and depression [35–37]. In view of this, we assume that anxiety can reduce college students’ sleep quality, which contributes to a high risk of depression.

Additional researches have shown that quality of life (QoL) may also contribute to the relationship between anxiety and depressive symptoms. First, studies have consistently shown that QoL is negatively affected by anxiety among medical students [38, 39]. Furthermore, there is evidence that shows a strong correlation between QoL and depression [40, 41]. Another national cross-sectional study used a multivariate analysis to show that higher mental summary scores were protective against depressive symptoms and, better physical health was associated with a decreased risk of depression; the same study also found that QoL was itself associated with depressive symptoms [42]. Moreover, another study predicted that the severity of anxiety symptoms is linked to greater health-related QoL impairment in college students; and that impaired mental health is more intensely correlated with depression than physical functioning impairment [43]. In light of the current evidence, we infer that anxiety symptoms will reduce QoL, and decreased QoL can lead to the occurrence of depressive symptoms among college students. Accordingly, we assumed that QoL could mediate anxiety and depression in our research.

Moreover, existing researches on sleep quality and QoL have found that college students with a history of sleep problems are more likely to experience a decreased QoL [44, 45]. Another study indicated that lower quality of sleep was significantly associated with other physical and psychological problems [46]. Furthermore, several researchers have identified a close association between sleep problems and QoL [47, 48].

In light of existing research and the previously mentioned gap in the literature, the goal of this study was to determine the association between anxiety and depression among college students. Furthermore, we examined the mediating role of sleep quality and QoL in the relationship between anxiety and symptoms of depression using a path analysis.

## Methods

### Participants

This cross-sectional study was conducted in November 2020 to evaluate the behaviors and mental health of college students. Participants were selected from two colleges located in Wuhu, Anhui Province: Wannan Medical College (a medical school) and Anhui Normal University (a non-medical school).

In the medical college, we randomly selected six majors (clinical medicine, nursing, preventive medicine, medical imaging, stomatology, and pharmacy), and then selected first-, second-, and third-year students according to the proportion of one-third in each major to complete the paper-and-pencil survey in small classes. Fourth and fifth year students were on probation or doing internship in accordance with the teaching requirements, and thus, did not participate in the survey. As per the inclusion criterion, undergraduate students engaged in school learning were considered. Students who were not in school or unwilling to participate in the survey were excluded. A total of 2400 questionnaires were distributed, of which 2318 were recovered (recovery rate: 96.58%), and 87 unqualified were eliminated; as such, the final medical school sample included 2231valid responses (effective rate: 96.25%).

Simultaneously, 600 students from some majors (the admission score of the college entrance examination is similar to that of Wannan Medical College) were randomly selected from Anhui Normal University to complete the paper-and-pencil survey. Among them, 582 were recovered (recovery rate: 97.00%), and 56 unqualified were eliminated; as such, 526 surveys were valid to be included in the sample (effective rate: 90.38%).

In sum, a total of 3000 questionnaires were distributed, 2900 were recovered (recovery rate: 96.67%), and 143 unqualified were eliminated; following this, a total of 2757 college students (1145 males and 1612 females) aged between 16 and 25 (mean = 19.07; SD = 1.14) participated in the study (with an efficacy rate of 95.07%).

This study was reviewed and approved by the Wannan Medical College’s Ethics Committee (No. LL-2020BH8003). Prior to the survey, all students were informed about the purpose of the study; after agreeing to voluntarily participate in it, each respondent signed a written informed consent form.

### Study design

Before distributing the formal survey, a pilot questionnaire was conducted and then improved and revised according to the feedback received. Furthermore, the investigators (graduate and undergraduate students majoring in preventive medicine) were then uniformly trained. During the formal investigation, the investigators explained the purpose of the survey to the counselor of the investigated classes. The investigators then informed the students of the purpose and significance of the survey, collected the informed consent forms, and issued the questionnaires. The investigators instructed the students to complete the questionnaires and then immediately submit them. In case of surveys with incorrect or missing information, the investigators were informed and the errors were corrected. The operation of data input was carried out by two people, and illogical data were simultaneously checked.

### Measures

The information collected included socio-demographic characteristics of the college students, such as gender, age, and whether they were an only child, as well as four measures regarding specific symptomatic conditions, namely, the 2-item General Anxiety Disorder (GAD-2), the 2-item Patient Health Questionnaire (PHQ-2), the Pittsburgh Sleep Quality Index (PSQI) and the Short-Form 36 Health Survey (SF-36).

The 2-item General Anxiety Disorder (GAD-2) [49, 50]. The GAD-2 is used to assess the frequency to which college students have experienced core anxiety symptoms during the past 2 weeks. It is comprised of 2 items (i.e. “Feeling nervous, anxious, or on edge” and “Not being able to stop or control worrying”). Responses are given on a 4-point Likert scale (not at all = 0; several days = 1; more than half the days = 2; nearly every day = 3), higher scores indicate more severe anxiety symptoms, and a score ≥ 3 is considered acceptable cut-point in screening for GAD. In the current study, the Cronbach’s alpha is 0.863.

The 2-item Patient Health Questionnaire (PHQ-2) [51, 52]. The PHQ-2 is used to assess the frequency to which college students have experienced depressed symptoms during the past 2 weeks. It is comprised of 2 items (i.e. “Little interest or pleasure in doing things” and “Feeling down, depressed, or hopeless”). Responses are also given on a 4-point Likert scale from 0 to 3. The total score ranges from 0 to 6, and a score ≥ 3 is considered reasonable cut-point in screening for PHQ. In the current study, the Cronbach’s alpha is 0.763.

The Pittsburgh Sleep Quality Index (PSQI) [53, 54]. The PSQI consists of 19 items and is used to evaluate sleep quality during the last month. This measure estimates 7 components of sleep, namely, subjective sleep quality, sleep latency, sleep duration, habitual sleep efficiency, sleep disturbance, use of sleeping medication, and daytime dysfunction. Each component is evaluated on a four-point Likert scale with the total scores ranging from 0 to 21, and the higher scores, the poorer sleep quality.

The Short-Form 36 Health Survey (SF-36) [55, 56]. The SF-36 is a valid measure to examine QoL among general and specific populations. It contains 36 items, including 8 dimensions, namely, Physical Functioning (PF), Role Physical (RP), Body Pain (BP), General Health (GH), Validity (VT), Social Functioning (SF), Role Emotional (RE), and Mental Health (MH). In addition, the first four dimensions constitute Physical Component Summary (PCS), and the last four dimensions constitute Mental Component Summary (MCS). Each item is scored separately and then converted to form a 0–100 scale, with greater scores illustrating the good quality of physical or mental health conditions. In the current study, the Cronbach’s alpha is 0.752.

### Statistical analysis

Analyses were performed using SPSS 26.0 software. We first conducted descriptive analyses of the socio-demographic characteristics among the college students. And, the Kolmogorov-Smirnov tests were performed to assess the normality distribution of anxiety, sleep quality, QoL and depression scores, respectively. The results concluded that anxiety, sleep quality, QoL and depression scores did not follow a normal distribution (*p*<0.05). Then, the Spearman’s rank correlation coefficients were used to evaluate the association between anxiety, sleep quality, QoL and depression scores. Finally, to examine whether sleep quality and QoL mediated the relationship between anxiety and depression, we conducted a mediation analysis with the SPSS PROCESS macro, version 3.4 (model 6), developed by Preacher and Hayes [57]. Additionally, the 95% confidence interval (CI) of mediating effects was estimated by 5000 samples to test the mediating role of sleep quality and QoL between anxiety and depression. Statistical significance level was set at *p*<0.05 (two-tailed).

### Common method biases

To eliminate the common method deviation caused by the questionnaire investigation, the Harman single-factor test was performed. The results of factor analysis found that there were 10 factors with characteristic roots were greater than 1, and the first common factor explained 17.70% (less than 40%) of the total variation. Hence, there were no obvious common method biases.

## Results

Table 1 shows that the demographic characteristics of the 2757 individuals who participated in this study. The mean age (SD) of the participants was 19.07 (1.14) years old. More than half (58.5%) of the participants were female, and 63.4% of the students had siblings. The median score (*P*_25_, *P*_75_) of the anxiety, depression, sleep quality and QoL of all the participants was 1.00 (0.00, 2.00), 1.00 (0.00, 2.00), 5.00 (5.00, 7.00) and 76.97 (65.06, 86.21), respectively.

**Table 1:** Demographic characteristics of study participants.

Characteristic	N or mean/median	% or SD or (*P*_25_, *P*_75_)
Age(years)	19.07	1.14
Gender		
Male	1145	41.5
Female	1612	58.5
Being the only child		
Yes	1008	36.6
No	1749	63.4
Sleep quality (PSQI global score)	5.00	(5.00, 7.00)
Quality of life (SF-36 score)	76.97	(65.06, 86.21)
PCS	84.75	(72.25, 93.00)
MCS	70.47	(56.25, 82.69)
Anxiety (GAD global score)	1.00	(0.00, 2.00)
Depression (PHQ global score)	1.00	(0.00, 2.00)

Note: PSQI, Pittsburgh Sleep Quality Index, SF-36, Short-Form 36 Health Survey, GAD-2, 2-item General Anxiety Disorder, PHQ-2, 2-item Patient Health Questionnaire

Results from the correlation analysis showed that anxiety was significantly associated with sleep quality (*r*_*s*_ = 0.413, *p*<0.001) and with QoL (*r*_*s*_ = -0.580, *p*<0.001) and with depression (*r*_*s*_ = 0.762, *p*<0.001). Sleep quality was also significantly associated with QoL (*r*_*s*_ = -0.491, *p*<0.001) and with depression (*r*_*s*_ = 0.443, *p*<0.001). Additionally, QoL and depression were also highly correlated (*r*_*s*_ = -0.601, *p*<0.001) (Table 2).

**Table 2:** Correlations between anxiety, sleep quality, quality of life, and depression.

	Anxiety	Sleep quality	Quality of life	Depression
Anxiety	1.000			
Sleep quality	0.413***	1.000		
Quality of life	-0.580***	-0.491***	1.000	
Depression	0.762***	0.443***	-0.601***	1.000

***p< 0.001 (two-tailed)

Multiple regression analysis showed that anxiety positively predicted sleep quality (*β* = 0.392, *p*<0.001). If anxiety and sleep quality were both taken as predictors, their effects on QoL were significant (*β* = -0.457, *p*<0.001 and -0.307, *p*<0.001). When anxiety, sleep quality, and QoL taken as predictors simultaneously, anxiety and sleep quality positively predicted depression (*β* = 0.622, 0.089; *p*<0.001), and QoL negatively predicted depression (*β* = -0.197, *p*<0.001) (Table 3).

**Table 3:** Regression analysis between variables.

Regression equation		Global fit index			Significance of regression coefficient	
Outcome variable	Predictor variable	*R*	*R* ^2^	*F*	*β*	*t*
Sleep quality	Anxiety	0.426	0.181	152.482	0.392	22.691***
Quality of life	Anxiety	0.641	0.411	384.159	-0.457	-28.616***
	Sleep quality				-0.307	-18.964***
Depression	Anxiety	0.798	0.637	803.789	0.622	43.576***
	Sleep quality				0.089	6.607***
	Quality of life				-0.197	-13.129***

Adjusted age, gender, and being the only child

***p< 0.001 (two-tailed)

**Fig 1:**
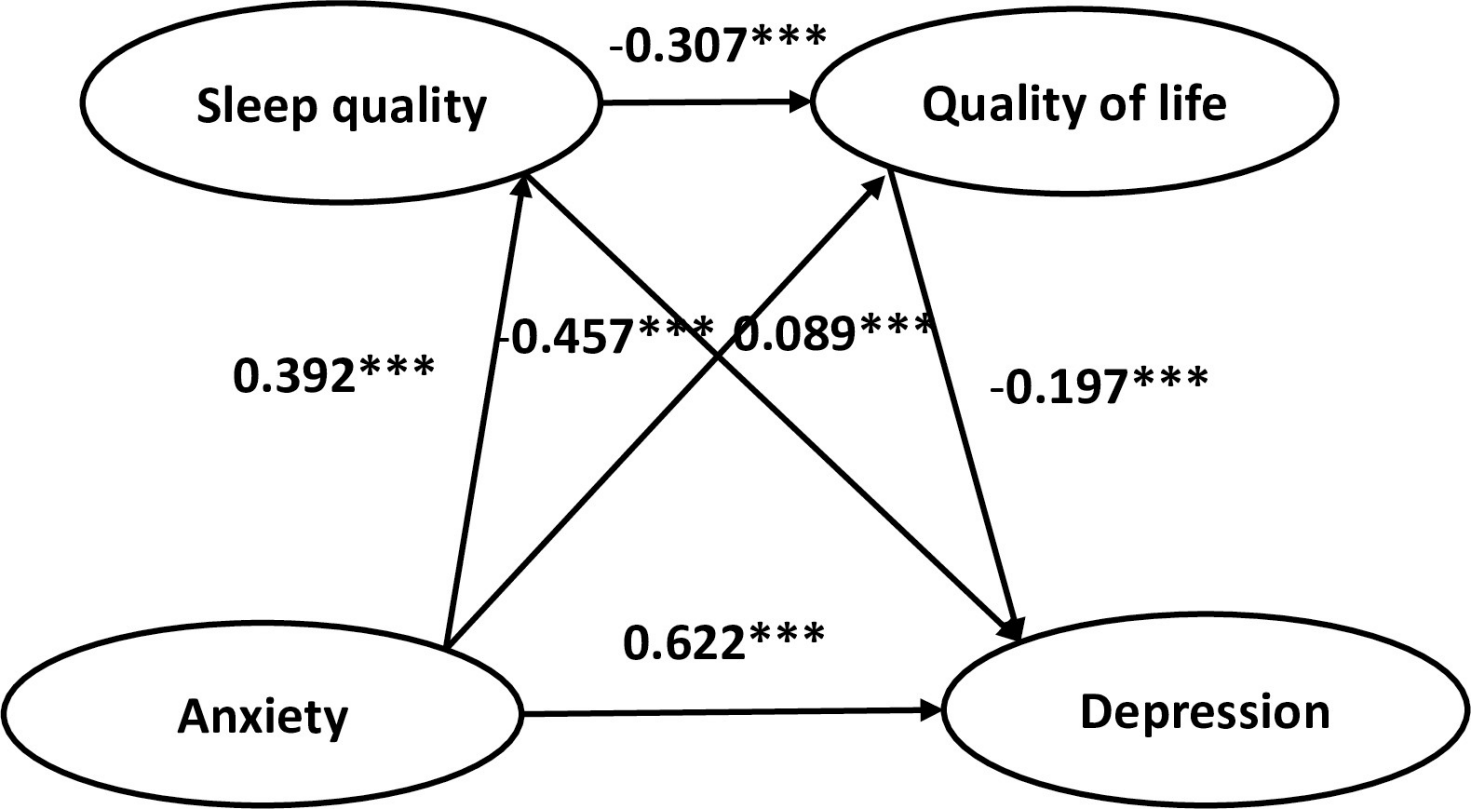
Model of mediating roles of sleep quality and quality of life between anxiety and depression. ***P<0.001.

As shown in Table 4, analyses of total indirect effects indicated that sleep quality and QoL served as partial mediating function in the relation between anxiety and depression (Effect = 0.138, SE = 0.010, 95%CI (0.119, 0.159)). The mediating effect accounted for 19.2% of the total effect (Effect = 0.719, SE = 0.011, 95%CI (0.697, 0.741)). Meanwhile, when tested separately, three mediating paths were significant: the indirect effects of anxiety on depression through sleep quality (Effect = 0.032, SE = 0.006, 95%CI (0.022, 0.044)), accounting for 4.5% of the total effect; the indirect effects of anxiety on depression through QoL (Effect = 0.084, SE = 0.008, 95%CI (0.069, 0.100)), accounting for 11.7% of the total effect; the indirect effects of anxiety on depression through sleep quality then QoL (Effect = 0.022, SE = 0.003, 95%CI (0.017, 0.027)), accounting for 3.0% of the total effect. The specific paths are presented in Fig 1.

**Table 4:** The mediating effect of sleep quality and QoL between anxiety and depression.

	Effect value	Boot SE	Boot CI lower	Boot CI upper	Relative mediation effect
Total effect	0.719	0.011	0.697	0.741	100.0%
Total indirect effect	0.138	0.010	0.119	0.159	19.2%
Indirect path 1	0.032	0.006	0.022	0.044	4.5%
Indirect path 2	0.084	0.008	0.069	0.100	11.7%
Indirect path 3	0.022	0.003	0.017	0.027	3.0%

Indirect path 1: the indirect effects of anxiety on depression through sleep quality

Indirect path 2: the indirect effects of anxiety on depression through QoL

Indirect path 3: the indirect effects of anxiety on depression through sleep quality then QoL

Considering QoL including both physical and mental aspects, PCS and MCS were separately tested whether can act as the mediators between anxiety and depression (Table 5). Both PCS and MCS can separately act as a mediator between anxiety and depression.

**Table 5:** The mediating effect of sleep quality and PCS between anxiety and depression.

	Effect value	Boot SE	Boot CI lower	Boot CI upper	Relative mediation effect
Total effect	0.719	0.011	0.697	0.741	100%
PCS					
Total indirect effect	0.093	0.009	0.077	0.111	12.9%
Indirect path 1	0.043	0.006	0.032	0.055	6.0%
Indirect path 2	0.039	0.006	0.028	0.051	5.4%
Indirect path 3	0.011	0.002	0.008	0.016	1.5%
MCS					
Total indirect effect	0.138	0.010	0.119	0.159	19.2%
Indirect path 1	0.034	0.006	0.025	0.046	4.7%
Indirect path 2	0.084	0.008	0.069	0.099	11.7%
Indirect path 3	0.020	0.002	0.016	0.025	2.8%

Indirect path 1: the indirect effects of anxiety on depression through sleep quality

Indirect path 2: the indirect effects of anxiety on depression through PCS/MCS

Indirect path 3: the indirect effects of anxiety on depression through sleep quality then QoL

## Discussion

To the best of our knowledge, this is the first large-scale study to investigate the relationship between anxiety, sleep quality, QoL, and symptoms of depression among college students; at the same time, the study aimed to estimate the mediating effects of sleep quality and QoL. The results showed that anxiety affected depression both directly and indirectly via sleep quality and QoL. Furthermore, sleep quality and QoL partially mediated the effects of anxiety on symptoms of depression. Specifically, anxiety was associated with poorer sleep quality, poorer sleep quality was associated with lower QoL, and a lower QoL predicted increased depression.

In accordance with previous research, we found that anxiety was associated with sleep quality, QoL, and depression. A previous study showed that anxiety may affect sleep quality through the gray matter volume of the right insula [28]. A study among medical students showed that students with anxiety symptoms experienced functional impairment in the psychological, social, and environmental domains of QoL [38]. Another study found that individuals with social anxiety disorder were more likely to score lower in the social, educational, and occupational domains, which might contribute to a poorer QoL [58]. One possible explanation for this strong association between anxiety and depression may be the similarity of their symptoms, which can include sleep problems, restlessness, and fatigue.

A different study indicated that the higher the anxiety level of college students, the stronger the excitability of the brain nerves, and the more difficult it is for individuals to fall asleep. In turn, the psychological pressure caused by struggling to fall asleep aggravated the degree of anxiety and finally led to individuals reducing their subjective evaluation of self-sleep quality [59]. Circadian rhythm may also play a vital role in the association between sleep quality and depression. For example, previous studies have demonstrated that circadian rhythm impacts affective disturbances, such as bipolar disorder and depression [60, 61]. During adolescence, the evening chronotype undergoes a dramatic change, which indicates vulnerability to emotional difficulties [62]. Additionally, the HPA axis is regarded as a physiological link between mental health and sleep problems [63–65]. A plausible explanation for the median effects of sleep quality may be that anxiety symptoms can disrupt college students’ normal sleep patterns, which results in a higher risk of depression.

Individuals with anxiety symptoms can experience both physical and psychological symptoms that interfere with school, work, and daily life [66]. The adverse effects of anxiety symptoms could result in low life satisfaction for individuals [67] and a productivity loss in society [68]. The underachievement and underperformance of college students may explain the negative effect of anxiety symptoms on QoL [69]. Negative emotions, such as anxiety, lead to a decline in mental health; and subsequently affect QoL, which, in turn, results in bad emotions, including depression.

College students worry about studying, success, and future plans, and they may experience the negative effects of anxiety as a result. Moreover, college students with anxiety symptoms often suffer from sleep problems due to circadian rhythms and the HPA axis. Since students with poor sleep quality are often lacking energy during the day, they may grow dependent on coffee, which makes them unable to fall asleep at night. Thus, they may kill time by using electronic devices at night. In short, coffee consumption and the use of electronic devices late at night will worsen their quality if sleep [48]. This will affect not only their quality of physical and mental life, but also \social relations, thereby reducing social QoL [45, 47, 70]. Irregular work, rest and insomnia will lead to a decline in sleep quality (which will lead to inattention while working and studying during the day), decrease in social activities (more likely to cause depression), and seriously affect the psychological state. These adverse psychological states further affect the QoL and daily life of college students [71].

The findings from the present study have significant clinical implications for psychologists who work with college students, as it provides evidence showing that higher anxiety levels lead to poorer sleep quality, lower QoL, and higher levels of depression. These findings provide preliminary support for targeting sleep quality and QoL in preventative interventions, particularly for college students with a higher propensity for anxiety. These results suggest that addressing sleep quality and QoL during the early stages of anxiety may prevent the onset of depression.

However, the current study has some limitations. First, the cross-sectional approach makes it impossible to infer causality. Future longitudinal studies are essential to confirm the predictive role of sleep quality and QoL in mediating the relationship between anxiety and depression among college students. Second, other confounding factors such as family situations, lifestyle, and eating habits were not explored in the current study.

In summary, our research found that sleep quality and QoL had a mediating effect on the relationship between anxiety and depression. These findings highlight the importance of early intervention among college students with anxiety, especially those with poorer sleep quality and lower QoL. Therefore, it is important for universities to take the initiative to identify students who are at a risk of developing psychological states and take steps to help support them from an early stage.

## Supporting information

S1 Data (SAV)

## References

[1] GBD 2019 Disease and Injuries Collaborators. Global burden of 369 diseases and injuries in 204 countries and territories, 1990–2019: a systematic analysis for the Global Burden of Disease Study 2019. Lancet. 2020; 396(10258): 1204–1222. doi: 10.1016/S0140-6736(20)30925-9 33069326 PMC7567026

[2] Wattick RA, Hagedorn RL, Olfert MD. Relationship between diet and mental health in a young adult Appalachian college population. Nutrients. 2018; 10(8): 957. 10.3390/nu10080957 PMC6115820 30044399

[3] Bayram N, Bilgel N. The prevalence and socio-demographic correlations of depression, anxiety and stress among a group of university students. Soc Psychiatry Psychiatr Epidemiol. 2008; 43(8): 667–672. doi: 10.1007/s00127-008-0345-x 18398558

[4] Kirsch DJ, Doerfler LA, Truong D. Mental health issues among college students: who gets referred for psychopharmacology evaluation? J Am Coll Health. 2015; 63(1): 50–56. doi: 10.1080/07448481.2014.960423 25222760

[5] Mao Y, Zhang N, Liu J, Zhu B, He R, Wang X. A systematic review of depression and anxiety in medical students in China. BMC Med Educ. 2019; 19(1): 327. doi: 10.1186/s12909-019-1744-2 31477124 PMC6721355

[6] Hysenbegasi A, Hass SL, Rowland CR. The impact of depression on the academic productivity of university students. J Ment Health Policy Econ. 2005; 8(3): 145–151. 16278502

[7] Khanam SJ, Bukhari SR. Depression as a predictor of academic performance in male and female university students. J Pak Psychiatr Soc. 2015; 12(2): 15–17.

[8] Asher BlackDeer A, Patterson Silver Wolf DA, Maguin E, Beeler-Stinn S. Depression and anxiety among college students: Understanding the impact on grade average and differences in gender and ethnicity. J Am Coll Health. 2021; 9: 1–12. doi: 10.1080/07448481.2021.1920954 34242525

[9] Bruffaerts R, Mortier P, Kiekens G, Auerbach RP, Cuijpers P, Demyttenaere K, et al. Mental health problems in college freshmen: Prevalence and academic functioning. J Affect Disord. 2018; 225: 97–103. doi: 10.1016/j.jad.2017.07.044 28802728 PMC5846318

[10] Boyraz G, Horne SG, Owens AC, Armstrong AP. Depressive Symptomatology and College Persistence among African American College Students. J Gen Psychol. 2016; 143(2): 144–60. doi: 10.1080/00221309.2016.1163251 27055080

[11] Shim EJ, Noh HL, Yoon J, Mun HS, Hahm BJ. A longitudinal analysis of the relationships among daytime dysfunction, fatigue, and depression in college students. J Am Coll Health. 2019; 67(1): 51–58. doi: 10.1080/07448481.2018.1462819 29652615

[12] Eisenberg D, Golberstein E, Hunt J. Mental Health and Academic Success in College. BE J Econ Anal Policy. 2011; 9(1): 40.

[13] Andrews B, Wilding JM. The relation of depression and anxiety to life-stress and achievement in students. Br J Psychol. 2004; 95(Pt 4): 509–521. doi: 10.1348/0007126042369802 15527535

[14] Adan RAH, van der Beek EM, Buitelaar JK, Cryan JF, Hebebrand J, Higgs S, et al. Nutritional psychiatry: towards improving mental health by what you eat. Eur Neuropsychopharmacol. 2019; 29(12): 1321–1332. doi: 10.1016/j.euroneuro.2019.10.011 31735529

[15] Hunt J, Eisenberg D. Mental health problems and help-seeking behavior among college students. J Adolesc Health. 2010; 46(1): 3–10. doi: 10.1016/j.jadohealth.2009.08.008 20123251

[16] Ngasa SN, Sama CB, Dzekem BS, Nforchu KN, Tindong M, Aroke D, et al. Prevalence and factors associated with depression among medical students in Cameroon: a cross-sectional study. BMC Psychiatry. 2017; 17(1):216. doi: 10.1186/s12888-017-1382-3 28599624 PMC5466797

[17] Aloufi MA, Jarden RJ, Gerdtz MF, Kapp S. Reducing stress, anxiety and depression in undergraduate nursing students: Systematic review. Nurse Educ Today. 2021; 102:104877. doi: 10.1016/j.nedt.2021.104877 33905898

[18] Ebert DD, Buntrock C, Mortier P, Auerbach R, Weisel KK, Kessler RC, et al. Prediction of major depressive disorder onset in college students. Depress Anxiety. 2019; 36(4): 294–304. doi: 10.1002/da.22867 30521136 PMC6519292

[19] Yu XD, Yu JC, Wu QF, Chen JY, Wang YC, Yan D, et al. The relationship among depression, anxiety, stress and addictive substance use behavior in 5 935 secondary vocational students. Zhonghua Yu Fang Yi Xue Za Zhi. 2017; 51(3): 226–231. Chinese. doi: 10.3760/cma.j.issn.0253-9624.2017.03.007 28260336

[20] Chang EC, Chang OD, Lucas AG, Li M, Beavan CB, Eisner RS, et al. Depression, Loneliness, and Suicide Risk among Latino College Students: A Test of a Psychosocial Interaction Model. Soc Work. 2019; 64(1): 51–60. doi: 10.1093/sw/swy052 30395325

[21] Zhang J, Liu Y, Sun L. Psychological strain and suicidal ideation: A comparison between Chinese and US college students. Psychiatry Res. 2017; 255: 256–262. doi: 10.1016/j.psychres.2017.05.046 28595148

[22] Guo L, Cao J, Cheng P, Shi D, Cao B, Yang G, et al. Moderate-to-Severe Depression Adversely Affects Lung Function in Chinese College Students. Front Psychol. 2020; 11: 652. doi: 10.3389/fpsyg.2020.00652 32351425 PMC7174779

[23] Shen Y, Zhang Y, Chan BSM, Meng F, Yang T, Luo X, et al. Association of ADHD symptoms, depression and suicidal behaviors with anxiety in Chinese medical college students. BMC Psychiatry. 2020; 20(1): 180. doi: 10.1186/s12888-020-02555-7 32321462 PMC7175542

[24] Kraft JD, Grant DM, White EJ, Taylor DL, Frosio KE. Cognitive mechanisms influence the relationship between social anxiety and depression among college students. J Am Coll Health.2021; 69(3): 245–251. doi: 10.1080/07448481.2019.1661844 31518208

[25] Beesdo K, Bittner A, Pine DS, Stein MB, Höfler M, Lieb R, et al. Incidence of social anxiety disorder and the consistent risk for secondary depression in the first three decades of life. Arch Gen Psychiatry. 2007; 64(8): 903–912. doi: 10.1001/archpsyc.64.8.903 17679635

[26] Johnson EO, Roth T and Breslau N. The association of insomnia with anxiety disorders and depression: Exploration of the direction of risk. J Psychiatr Res. 2006; 40(8):700–708. doi: 10.1016/j.jpsychires.2006.07.008 16978649

[27] Sivertsen B, Krokstad S, Øverland S, Mykletun A. The epidemiology of insomnia: associations with physical and mental health. The HUNT-2 study. J Psychosom Res. 2009; 67(2): 109–116. doi: 10.1016/j.jpsychores.2009.05.001 19616137

[28] Yin H, Zhang L, Li D, Xiao L, Cheng M. The gray matter volume of the right insula mediates the relationship between symptoms of depression/anxiety and sleep quality among college students. J Health Psychol. 2021; 26(7): 1073–1084. doi: 10.1177/1359105319869977 31411064

[29] Cahuas A, He Z, Zhang Z, Chen W. Relationship of physical activity and sleep with depression in college students. J Am Coll Health. 2020; 68(5): 557–564. doi: 10.1080/07448481.2019.1583653 30908132

[30] Zou P, Wang X, Sun L, Liu K, Hou G, Yang W, et al. Poorer sleep quality correlated with mental health problems in college students: A longitudinal observational study among 686 males. J Psychosom Res.2020; 136: 110177. doi: 10.1016/j.jpsychores.2020.110177 32623194

[31] Zou L, Wu X, Tao S, Xu H, Xie Y, Yang Y, et al. Mediating Effect of Sleep Quality on the Relationship Between Problematic Mobile Phone Use and Depressive Symptoms in College Students. Front Psychiatry. 2019; 10: 822. doi: 10.3389/fpsyt.2019.00822 31798473 PMC6865206

[32] Williams AB, Dzierzewski JM, Griffin SC, Lind MJ, Dick D, Rybarczyk BD. Insomnia Disorder and Behaviorally Induced Insufficient Sleep Syndrome: Prevalence and Relationship to Depression in College Students. Behav Sleep Med. 2020; 18(2): 275–286. doi: 10.1080/15402002.2019.1578772 30789063 PMC6814500

[33] van Dalfsen JH, Markus CR. Interaction between 5-HTTLPR genotype and cognitive stress vulnerability on sleep quality: effects of sub-chronic tryptophan administration. Int J Neuropsychopharmacol. 2015; 18(3): pyu057. doi: 10.1093/ijnp/pyu057 25644221 PMC4360245

[34] Wang FZ, Wang YJ. Regulation of locus coeruleus and central noradrenergic system on hypothalamic pituitary adrenocorticak axis. Progress in Physiological Sciences. 1982; (3): 208–212. Chinese. 6294824

[35] Mann JJ, Huang YY, Underwood MD, Kassir SA, Oppenheim S, Kelly TM, et al. A serotonin transporter gene promoter polymorphism (5-HTTLPR) and prefrontal cortical binding in major depression and suicide. Arch Gen Psychiatry. 2000; 57(8): 729–738. doi: 10.1001/archpsyc.57.8.729 10920459

[36] Lesch KP, Bengel D, Heils A, Sabol SZ, Greenberg BD, Petri S, et al. Association of anxiety-related traits with a polymorphism in the serotonin transporter gene regulatory region. Science. 1996; 274(5292):1527–1531. doi: 10.1126/science.274.5292.1527 8929413

[37] Blake MJ, Trinder JA, Allen NB. Mechanisms underlying the association between insomnia, anxiety, and depression in adolescence: Implications for behavioral sleep interventions. Clin Psychol Rev. 2018; 63: 25–40. doi: 10.1016/j.cpr.2018.05.006 29879564

[38] Gan GG, Yuen Ling H. Anxiety, depression and quality of life of medical students in Malaysia. Med J Malaysia. 2019; 74(1): 57–61. 30846664

[39] Dyrbye LN, Thomas MR, Massie FS, Power DV, Eacker A, Harper W, et al. Burnout and suicidal ideation among U.S. medical students. Ann Intern Med. 2008; 149(5): 334–341. doi: 10.7326/0003-4819-149-5-200809020-00008 18765703

[40] Paro HB, Morales NM, Silva CH, Rezende CH, Pinto RM, Morales RR, et al. Health-related quality of life of medical students. Med Educ. 2010; 44(3): 227–235. doi: 10.1111/j.1365-2923.2009.03587.x 20444053

[41] de Leval N. Quality of life and depression: symmetry concepts. Qual Life Res. 1999; 8(4): 283–291. doi: 10.1023/a:1008970317554 10472160

[42] Zhong X, Liu Y, Pu J, Tian L, Gui S, Song X, et al. Depressive symptoms and quality of life among Chinese medical postgraduates: a national cross-sectional study. Psychol Health Med. 2019; 24(8): 1015–1027. doi: 10.1080/13548506.2019.1626453 31179736

[43] Jenkins PE, Ducker I, Gooding R, James M, Rutter-Eley E. Anxiety and depression in a sample of UK college students: a study of prevalence, comorbidity, and quality of life. J Am Coll Health. 2021; 69(8): 813–819. doi: 10.1080/07448481.2019.1709474 31995452

[44] Sarıarslan HA, Gulhan YB, Unalan D, Basturk M, Delibas S. The relationship of sleep problems to life quality and depression. Neurosciences. 2015; 20(3): 236–242. doi: 10.17712/nsj.2015.3.20150157 26166591 PMC4710343

[45] Kwon SJ, Kim Y, Kwak Y. Relationship of sleep quality and attention deficit hyperactivity disorder symptoms with quality of life in college students. J Am Coll Health. 2020; 68(5): 536–542. doi: 10.1080/07448481.2019.1583650 30908170

[46] Lund HG, Reider BD, Whiting AB, Prichard JR. Sleep patterns and predictors of disturbed sleep in a large population of college students. J Adolesc Health. 2010; 46(2): 124–32. doi: 10.1016/j.jadohealth.2009.06.016 20113918

[47] Sandadi S, Frasure HE, Broderick MJ, Waggoner SE, Miller JA, von Gruenigen VE. The effect of sleep disturbance on quality of life in women with ovarian cancer. Gynecol Oncol. 2011; 123(2): 351–355. doi: 10.1016/j.ygyno.2011.07.028 21855973

[48] Rezaei O, Mokhayeri Y, Haroni J, Rastani MJ, Sayadnasiri M, Ghisvand H, et al. Association between sleep quality and quality of life among students: a cross sectional study. Int J Adolesc Med Health. 2017; 32(2) /j/ijamh.2020.32.issue-2/ijamh-2017-0111/ijamh-2017-0111.xml. doi: 10.1515/ijamh-2017-0111 28915114

[49] Kroenke K, Spitzer RL, Williams JB, Monahan PO, Lowe B. Anxiety disorders in primary care: prevalence, impairment, comorbidity, and detection. Ann Intern Med. 2007; 146(5): 317–325. doi: 10.7326/0003-4819-146-5-200703060-00004 17339617

[50] Luo Z, Li Y, Hou Y, Zhang H, Liu X, Qian X, et al. Adaptation of the two-item generalized anxiety disorder scale (GAD-2) to Chinese rural population: A validation study and meta-analysis. Gen Hosp Psychiatry. 2019; 60: 50–56. doi: 10.1016/j.genhosppsych.2019.07.008 31326672

[51] Kroenke K, Spitzer RL, Williams JB. The Patient Health Questionnaire-2: validity of a two-item depression screener. Med Care. 2003; 41(11): 1284–1292. doi: 10.1097/01.MLR.0000093487.78664.3C 14583691

[52] Jessica A Gold Xinran Hu, Huang Gan, Li Wan-Zhen, Wu Yi-Fan, Gao Shan, et al. Medical student depression and its correlates across three international medical schools. World J Psychiatry, 2019; 9(4): 65–77. doi: 10.5498/wjp.v9.i4.65 31799151 PMC6885454

[53] Buysse DJ, Reynolds CF 3rd, Monk TH, Berman SR, Kupfer DJ. The Pittsburgh Sleep Quality Index: A new instrument for psychiatric practice and research. Psychiatry Res. 1989; 28(2): 193–213. doi: 10.1016/0165-1781(89)90047-4 2748771

[54] Tao S, Wu X, Zhang Y, Zhang S, Tong S, Tao F. Effects of Sleep Quality on the Association between Problematic Mobile Phone Use and Mental Health Symptoms in Chinese College Students. Int J Environ Res Public Health. 2017;14(2):185. doi: 10.3390/ijerph14020185 28216583 PMC5334739

[55] Qiu Y, Yao M, Guo Y, Zhang X, Zhang S, Zhang Y, et al. Health-Related Quality of Life of Medical Students in a Chinese University: A Cross-Sectional Study. Int J Environ Res Public Health. 2019; 16(24): 5165. 10.3390/ijerph16245165 PMC6950113 31861231

[56] Li lu, Wang hongmei, Shen yi. Development and psychometric tests of a Chinese version of the SF-36 Health Survey Scales. Chin J Prevent Med. 2002; 36(2): 38–42. 12410965

[57] Hayes AF. PROCESS: A versatile computational tool for observed variable mediation, moderation, and conditional process modeling. 2012.

[58] Dryman MT, Gardner S, Weeks JW, Heimberg RG. Social anxiety disorder and quality of life: How fears of negative and positive evaluation relate to specific domains of life satisfaction. J Anxiety Disord. 2016; 38: 1–8. doi: 10.1016/j.janxdis.2015.12.003 26709747

[59] Yan Zhang, Fei Li, Wen-hua Zhou, Bing-kun Li. Meta-analysis of the relationship between sleep quality and the mental health among Chinese college students. Chin J Sch Health. 2014: 35(3): 381–384. Chinese.

[60] Wulff K, Gatti S, Wettstein JG, Foster RG. Sleep and circadian rhythm disruption in psychiatric and neurodegenerative disease. Nat rev Neurosci. 2010; 11(8): 589–99. doi: 10.1038/nrn2868 20631712

[61] Urrila AS, Paunio T, Palomaki E, Marttunen M. Sleep in adolescent depression: physiological perspectives. Acta physiol. 2015; 213(4): 758–77. doi: 10.1111/apha.12449 25561272

[62] Dagys N, McGlinchey EL, Talbot LS, Kaplan KA, Dahl RE, Harvey AG. Double trouble? The effects of sleep deprivation and chronotype on adolescent affect. J child psycho psychiatry. 2012: 53(6): 660–667. doi: 10.1111/j.1469-7610.2011.02502.x 22188424 PMC3311740

[63] Guerry JD, Hastings PD. In search of HPA axis dysregulation in child and adolescent depression. Clin Child Fam Psychol Rev. 2011; 14(2): 135–160. doi: 10.1007/s10567-011-0084-5 21290178 PMC3095794

[64] Lopez-Duran NL, Kovacs M, George CJ. Hypothalamic-pituitary-adrenal axis dysregulation in depressed children and adolescents: a meta-analysis. Psychoneuroendocrinology. 2009; 34(9): 1272–1283. doi: 10.1016/j.psyneuen.2009.03.016 19406581 PMC2796553

[65] Rhebergen D, Korten NC, Penninx BW, Stek ML, van der Mast RC, Oude Voshaar R, et al. Hypothalamic-pituitary-adrenal axis activity in older persons with and without a depressive disorder. Psychoneuroendocrinology. 2015; 51: 341–350. doi: 10.1016/j.psyneuen.2014.10.005 25462906

[66] Remes O, Brayne C, van der Linde R, Lafortune L. A systematic review of reviews on the prevalence of anxiety disorders in adult populations. Brain Behav. 2016; 6(7): e00497. doi: 10.1002/brb3.497 27458547 PMC4951626

[67] Mahmoud JS, Staten R, Hall LA, Lennie TA. The relationship among young adult college students’ depression, anxiety, stress, demographics, life satisfaction, and coping styles. Issues Ment Health Nurs. 2012; 33(3):149–56. doi: 10.3109/01612840.2011.632708 22364426

[68] Sado M, Takechi S, Inagaki A, Fujisawa D, Koreki A, Mimura M, et al. Cost of anxiety disorders in Japan in 2008: a prevalence-based approach. BMC Psychiatry. 2013; 13:338. doi: 10.1186/1471-244X-13-338 24350841 PMC3878364

[69] Alsamghan AS. Social Anxiety Symptoms and Quality of Life of Secondary School Students of Abha, Saudi Arabia. J Genet Psychol. 2021; 182(1): 18–30. doi: 10.1080/00221325.2020.1837065 33135586

[70] Awadalla NJ, Mahfouz AA, Shehata SF, Al Thibiait SA, Aljihani AH, Hafez SM, et al. Sleep hygiene, sleep-related problems, and their relations with quality of life in a primary-care population in southwest Saudi Arabia. J Family Med Prim Care. 2020; 9(6): 3124–3130. doi: 10.4103/jfmpc.jfmpc_525_20 32984184 PMC7491796

[71] Li J, Liu W, Yu BL, Li XL, Cao XJ. Canonical correlation analysis on sleep quality and quality of life among college students. Acta Universitatis Medicinalis Anhui. 2009; 54(6): 942–945.Chinese.

